# (NH_4_)Mg(HSO_4_)(SO_4_)(H_2_O)_2_ and NaSc(CrO_4_)_2_(H_2_O)_2_, two crystal structures com­prising kröhnkite-type chains, and the temperature-induced phase transition (NH_4_)Mg(HSO_4_)(SO_4_)(H_2_O)_2_ 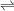 (NH_4_)MgH(SO_4_)_2_(H_2_O)_2_

**DOI:** 10.1107/S2053229621001650

**Published:** 2021-02-22

**Authors:** Matthias Weil, Uwe Kolitsch

**Affiliations:** a Institute for Chemical Technologies and Analytics, Division of Structural Chemistry, TU Wien, Getreidemarkt 9/164-SC, A-1060 Vienna, Austria; b Mineralogisch-Petrographische Abt., Naturhistorisches Museum, Burgring 7, A-1010 Wien, Austria; c Institut für Mineralogie und Kristallographie, Universität Wien, Althanstrasse 14, A-1090 Wien, Austria

**Keywords:** ammonium magnesium sulfate, strong hydrogen bond, sodium scandium chromate, tetra­hedral-octa­hedral chains, kröhnkite-type chains, phase transition, crystal structure, IR spectroscopy

## Abstract

The crystal structure of the mineral kröhnkite, Na_2_Cu(SO_4_)_2_(H_2_O)_2_, contains infinite chains com­posed of [CuO_4_(OH_2_)_2_] octa­hedra corner-linked with SO_4_ tetra­hedra. Such or similar tetra­hedral–octa­hedral ‘kröhnkite-type’ chains are present in the crystal structures of numerous com­pounds with the com­position *A_n_M*(*X*O_4_)_2_(H_2_O)_2_. The title com­pounds, (NH_4_)Mg(HSO_4_)(SO_4_)(H_2_O)_2_, ammonium magnesium hydrogen sulfate sulfate dihydrate, and NaSc(CrO_4_)_2_(H_2_O)_2_, sodium scandium bis­(chromate) dihydrate, are members of the large family with such kröhnkite-type chains. At 100 K, (NH_4_)Mg(HSO_4_)(SO_4_)(H_2_O)_2_ has an unprecedented triclinic crystal structure and contains [MgO_4_(OH_2_)_2_] octa­hedra linked by SO_3_(OH) and SO_4_ tetra­hedra into chains extending parallel to [10]. Adjacent chains are linked by very strong hydrogen bonds between SO_3_(OH) and SO_4_ tetra­hedra into layers parallel to (111). Ammonium cations and water mol­ecules connect adjacent layers through hydrogen-bonding inter­actions of medium-to-weak strength into a three-dimensional network. (NH_4_)Mg(HSO_4_)(SO_4_)(H_2_O)_2_ shows a reversible phase transition and crystallizes at room temperature in structure type **E** in the classification scheme for structures with kröhnkite-type chains, with half of the unit-cell volume for the resulting triclinic cell, and with disordered H atoms of the ammonium tetra­hedron and the H atom between two symmetry-related sulfate groups. IR spectroscopic room-temperature data for the latter phase are provided. Monoclinic NaSc(CrO_4_)_2_(H_2_O)_2_ adopts structure type **F1** in the classification scheme for structures with kröhnkite-type chains. Here, [ScO_4_(OH_2_)_2_] octa­hedra (point group symmetry ) are linked by CrO_4_ tetra­­hedra into chains parallel to [010]. The Na^+^ cations (site symmetry 2) have a [6 + 2] coordination and connect adjacent chains into a three-dimensional frame­work that is consolidated by medium–strong hydrogen bonds involving the water mol­ecules. Qu­anti­tative structural com­parisons are made between NaSc(CrO_4_)_2_(H_2_O)_2_ and its isotypic Na*M*(CrO_4_)_2_(H_2_O)_2_ (*M* = Al and Fe) analogues.

## Abstract 2

Both dihydrates (NH_4_)Mg(HSO_4_)(SO_4_)(H_2_O)_2_ and NaSc(CrO_4_)_2_(H_2_O)_2_ com­prise kröhnkite-type tetra­hedral–octa­hedral chains made up of {SO_4_/SO_3_(OH),[MgO_4_(OH_2_)_2_]} and {CrO_4_,[ScO_4_(OH_2_)_2_]} building units, respectively. (NH_4_)Mg(HSO_4_)(SO_4_)(H_2_O)_2_ shows a reversible phase transition between 100 and 296 K.

## Introduction   

Compounds com­prising tetra­hedral oxoanions (*X*O_4_) and two types of cations, *viz*. a larger cation *A* and a smaller cation *M*, often exist as dihydrates with the general formula *A_n_M*(*X*O_4_)_2_(H_2_O)_2_ (*n* = 1 or 2) when crystallized from aqueous solutions or under hydro­thermal conditions. Irrespective of the chemical nature of *A*, *M* or *X*, the crystal structures of *A_n_M*(*X*O_4_)_2_(H_2_O)_2_ com­pounds frequently com­prise infinite chains com­posed of more or less distorted [*M*O_4_(OH_2_)_2_] octa­hedra corner-linked by *X*O_4_ tetra­hedra, a structural motif that is known from the mineral kröhnkite [Na_2_Cu(SO_4_)_2_(H_2_O)_2_; Dahlman, 1952 ▸]. An astonishingly large number of natural and synthetic hydrated oxysalts with this formula type is known to contain such ‘kröhnkite-type’ chains in their crystal structures. The widespread occurence of this motif is associated with its flexible nature and assemblies of the corner-sharing octa­hedral–tetra­hedral building units within a chain.

Reviews on natural and synthetic com­pounds with kröhnkite-type chains were given in four subsequent reports (Fleck *et al.*, 2002*a* ▸; Fleck & Kolitsch, 2003 ▸; Kolitsch & Fleck, 2005 ▸, 2006 ▸). In general, com­pounds with the com­position *A_n_M*(*X*O_4_)_2_(H_2_O)_2_, where *A* = Na^I^, K^I^, Rb^I^, Cs^I^, NH_4_, H^I^, Ca^II^ or Sr^II^; *M* = Mg^II^, Cr^II^, Mn^II^, Fe^II^, Co^II^, Ni^II^, Cu^II^, Zn^II^, Cd^II^, Al^III^, Fe^III^, Sc^III^, In^III^ or Tl^III^ and *X* = P^V^, As^V^, S^VI^, Se^VI^, Cr^VI^, Mo^VI^ or W^VI^, containing kröhnkite-type chains, can be subdivided into eight major structure types denoted as **A**–**H**, for which more than 70 representatives are known up to date. Table 1 ▸ com­piles the most important parameters for these structure types, based on all representatives reported until the end of 2020, including the new type **E1** described herein.

We report here two new representatives of com­pounds with kröhnkite-type chains, *viz*. (NH_4_)Mg(HSO_4_)(SO_4_)(H_2_O)_2_ and NaSc(CrO_4_)_2_(H_2_O)_2_.

## Experimental   

### Synthesis and crystallization   

#### (NH_4_)Mg(HSO_4_)(SO_4_)(H_2_O)_2_   

A stoichiometric mixture of MgSO_4_(H_2_O)_7_, TeO_2_ and KOH (ratio 2:1:2 mmol; all reagents from Merck) was placed in a Teflon container with 6 ml capacity that was filled to approximately two-thirds of its volume with water. The container was closed, placed in a steel autoclave and heated at 480 K under autogenous pressure for 4 d. After slow cooling to room temperature within 1 d, the colourless reaction product was filtered off, washed with water and ethanol, and was dried in air. Inspection under a polarizing microscope revealed a microcrystalline solid with only very few crystals visible (diameter ≃ 0.1 mm). Powder X-ray diffraction (PXRD) of the bulk revealed spiro­ffite-type Mg_2_Te_3_O_8_ (Lin *et al.*, 2013 ▸) as the main product, and MgTe_2_O_5_ (Weil, 2005 ▸) as a minor product. The grown crystals correspond to the title com­pound. Structure refinement showed NH_4_^+^ cations present in the structure. The source of ammonium remains unclear; most probably, ammonium cations were left in the cracks of the Teflon container from previous reactions in ammonia solution.

For a directed synthesis of (NH_4_)Mg(HSO_4_)(SO_4_)(H_2_O)_2_, equimolar aqueous solutions of NH_4_HSO_4_ and MgSO_4_(H_2_O)_7_ were mixed at room temperature and stirred for homogeneity. The used NH_4_HSO_4_ was freshly prepared by slowly adding concentrated ammonia solution to concentrated sulfuric acid in stoichiometric amounts and recrystallization of the colourless product from water; its purity was checked by PXRD. The mixed NH_4_HSO_4_ and MgSO_4_ solutions were evaporated to dryness at 353 K in a drying oven and also much more slowly at room temperature. Semi-qu­anti­taive phase analysis using the Rietveld method with *HighScore Plus* (Degen *et al.*, 2014 ▸) revealed a phase mixture of (NH_4_)MgH(SO_4_)_2_(H_2_O)_2_ and synthetic boussingaultite [(NH_4_)_2_Mg(SO_4_)_2_(H_2_O)_6_] in a ratio of ≃94:6 wt% for the sample dried at 353 K, and in a ratio of 91:9 wt% for the sample dried at room temperature.

A mid-range IR spectrum was recorded at room temperature for selected crystals of (NH_4_)MgH(SO_4_)_2_(H_2_O)_2_ in the attenuated total reflectance (ATR) technique in the range 4000–450 cm^−1^ on a PerkinElmer Spectrum Two FT–IR spec­trometer with a UATR accessory (diamond detector crystal) attached.

#### NaSc(CrO_4_)_2_(H_2_O)_2_   

Small tabular orange crystals with a rhombus-shaped outline crystallized at room tem­pera­ture from an acidic aqueous solution (pH about 4) containing dissolved reagent-grade Na_2_CO_3_ (Merck), Sc_2_O_3_ (99.99%, alphametall, Germany) and reagent-grade CrO_3_ (Merck). The crystals were often arranged in radiating clusters. They were associated with pale orange–yellow blade-shaped crystals of Na_2_Cr_2_O_7_·2H_2_O (Casari *et al.*, 2007 ▸).

### Refinement   

Crystal data, data collection and structure refinement details are summarized in Table 2 ▸.

**Figure 1:**
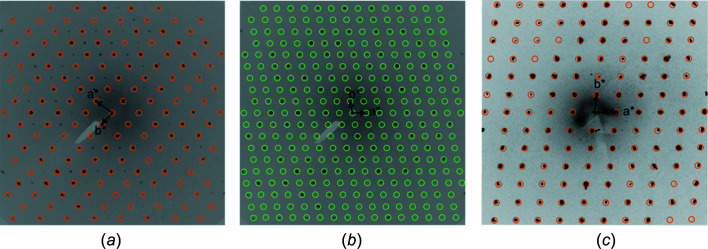
The *hk*1 plane of reciprocal space of (NH_4_)Mg(HSO_4_)(SO_4_)(H_2_O)_2_ [100 K data in parts (*a*)/(*b*)] and of (NH_4_)MgH(SO_4_)_2_(H_2_O)_2_ [296 K data in part (*c*)] reconstructed from CCD data. (*a*) The small cell with matching reflections is marked with orange circles; no superstructure reflections are visible. (*b*) The actual supercell with matching reflections is marked with green circles. (*c*) The actual small cell is marked with orange circles without superstructure reflections.

For the refinement of (NH_4_)Mg(HSO_4_)(SO_4_)(H_2_O)_2_ (100 K data), all H atoms were located from difference Fourier maps and were refined freely. Reflections 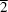
03, 102, 100 and 230 were obstructed by the beam stop and were therefore omitted from the refinement. For the structure analysis of (NH_4_)MgH(SO_4_)_2_(H_2_O)_2_ (296 K data), a different crystal was measured (resulting in a different orienting matrix; see Fig. 1 ▸). For refinement, the starting coordinates and labelling of atoms were adapted from the isotypic Fe com­pound (Heinicke *et al.*, 2004 ▸). In this structure, the H atoms (H1*A*–H1*D*) bonded to N atoms are all disordered over two equally occupied sites. The H1O atom located between two symmetry-related SO_4_ tetra­hedra was clearly discernible from a difference Fourier map; it is disordered across the inversion centre with an occupancy of 0.5 for the two H-atom sites. All H atoms in this structure were refined freely.

For refinement of NaSc(CrO_4_)_2_(H_2_O)_2_, the coordinates and labelling of atoms were taken from isotypic NaFe(CrO_4_)_2_(H_2_O)_2_ (Hardy & Gravereau, 1970 ▸). The H atoms of the water mol­ecule were located from a difference Fourier map and were refined with a constraint of O—H = 0.90 ± 0.03 Å.

## Results and discussion   

### (NH_4_)Mg(HSO_4_)(SO_4_)(H_2_O)_2_   

#### Structure analysis   

**Figure 2:**
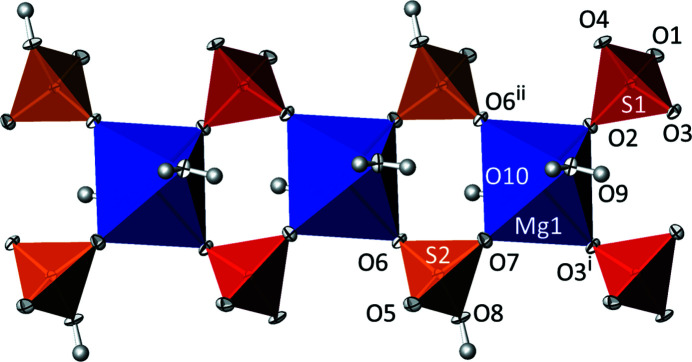
The kröhnkite-type chain in the crystal structure of (NH_4_)Mg(HSO_4_)(SO_4_)(H_2_O)_2_ at 100 K. [MgO_4_(OH_2_)_2_] octa­hedra are blue, SO_3_(OH) tetra­hedra are orange and SO_4_ tetra­hedra are red. Displacement ellipsoids are drawn at the 74% probability level and H atoms are given as grey spheres of arbitrary radius. [Symmetry codes: (i) −*x*, −*y* + 1, −*z* + 1; (ii) −*x* + 1, −*y*, −*z* + 1.]

**Figure 3:**
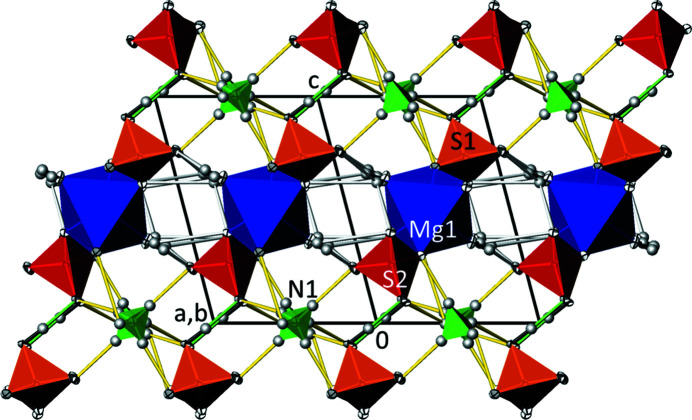
The crystal structure of (NH_4_)Mg(HSO_4_)(SO_4_)(H_2_O)_2_ at 100 K in a projection along [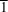
10]. [MgO_4_(OH_2_)_2_] octa­hedra are blue, SO_4_ tetra­hedra are red, SO_3_(OH) tetra­hedra are orange and ammonium groups are green. The strong hydrogen bond between the SO_3_(OH) and SO_4_ tetra­hedra is indicated by green lines, hydrogen bonds involving water mol­ecules by white lines and those involving the ammonium cations by yellow lines. Displacement ellipsoids are drawn at the 74% probability level.

(NH_4_)Mg(HSO_4_)(SO_4_)(H_2_O)_2_ was obtained serendipitously from a hydro­thermal synthesis intended to crystallize a com­pound in the system Mg–S^VI^–Te^IV^–O–H (Weil & Shirkhanlou, 2017 ▸). A subsequently performed directed synthesis yielded this material in >90% yield by evaporation of an aqueous solution containing equimolar amounts of NH_4_HSO_4_ and MgSO_4_.

(NH_4_)Mg(HSO_4_)(SO_4_)(H_2_O)_2_ is the fourth com­pound in the NH_3_–MgO–SO_3_–H_2_O system. The three other known members are the two minerals boussingaultite, *i.e.* (NH_4_)_2_Mg(SO_4_)_2_(H_2_O)_6_ (Maslen *et al.*, 1988 ▸), and efremovite, *i.e.* (NH_4_)_2_Mg_2_(SO_4_)_3_ (Shcherbakova & Bazhenova, 1989 ▸), and synthetic (NH_4_)_2_Mg_3_(OH)_2_(SO_4_)_3_(H_2_O)_2_ (Marri *et al.*, 2017 ▸). Monoclinic boussingaultite is a representative of the picromerite group and crystallizes isotypically with many other synthetic *A* ^I^_2_ *M* ^II^(*X*O_4_)_2_(H_2_O)_6_ com­pounds (*A* ^I^ = NH_4_, K, Rb, Cs or Tl; *M* ^II^ = Mg, V, Cr, Mn, Fe, Co, Ni, Cu, Zn or Cd; *X* = S, Se or Cr), commonly known as Tutton’s salts [crystal structure first determined by Hofmann (1931 ▸)]. Efremovite adopts the cubic langbeinite structure type (Zemann & Zemann, 1957 ▸), and ortho­rhom­bic (NH_4_)_2_Mg_3_(OH)_2_(SO_4_)_3_(H_2_O)_2_ is isotypic with its cadmium analogue (NH_4_)_2_Cd_3_(OH)_2_(SO_4_)_3_(H_2_O)_2_ (Yin, 2011 ▸).

(NH_4_)Mg(HSO_4_)(SO_4_)(H_2_O)_2_ is an unprecedented mem­ber within the family of com­pounds with kröhnkite-type chains and crystallizes in a unique structure type at 100 K, here denoted as **E1** in order to conform to the classification of compounds with kröhnkite-type chains (Fleck *et al.*, 2002*a* ▸; Table 1 ▸). All atoms in the triclinic structure are situated on general positions. [MgO_4_(OH_2_)] octa­hedra are corner-linked by SO_3_(OH) and SO_4_ tetra­hedra into chains running parallel to [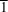
10] (Fig. 2 ▸). Adjacent chains are joined by hydrogen bonds between hydrogen sulfate and sulfate tetra­hedra into sheets extending parallel to (111). Ammonium cations, situated between the sheets, and water mol­ecules are also involved in hydrogen bonding and consolidate the three-dimensional network (Fig. 3 ▸).

The Mg—O bond lengths in the [MgO_4_(OH_2_)_2_] octa­hedron scatter only slightly [range 2.0382 (9)–2.0715 (9) Å; Table 3 ▸], with the two *trans*-aligned water mol­ecules (O9 and O19) in the axial positions. The mean Mg—O distance of 2.061 Å fits well into the grand mean value of 2.09 (6) Å for six-coordinate Mg^II^ (Gagné & Hawthorne, 2016 ▸). The SO_4_ tetra­hedron (centred by atom S1) is slightly distorted, with bond lengths and angles in the ranges 1.4659 (9)–1.4901 (9) Å (mean 1.476 Å) and 106.87 (5)–111.52 (5)° (mean 109.5°), respectively. The bond-length values are in very good agreement with those given in a review on the sulfate group, for which the grand mean S—O distance is 1.473 Å, with minimum and maximum S—O distances of 1.430 and 1.501 Å, respectively (Hawthorne *et al.*, 2000 ▸). The longest bond in the S1O_4_ tetra­hedron is that to atom O4, acting as an acceptor atom for a hydrogen bond involving the OH group of the hydrogen sulfate group. The corresponding S2O_3_(OH) tetra­hedron shows the typical S—O bond-length distribution where the bond to the OH group (O8) is considerably elongated. The S2—O8 bond of 1.5474 (9) Å is about 0.09 Å longer than the mean bond length (1.456 Å) of the remaining three bonds, in good agreement with other structures com­prising a hydrogen sulfate anion, *e.g*. Mg(HSO_4_)_2_(H_2_O) (Worzala *et al.*, 1991 ▸) or Th(HSO_4_)_2_(SO_4_) (Betke & Wickleder, 2012 ▸). In the magnesium com­pound, with its two independent SO_3_(OH) tetra­hedra, mean values of 1.448 Å for the S—O and 1.550 Å for the S—OH bond lengths are found, and for the thorium com­pound, the corresponding mean values are 1.452 and 1.533 Å, respectively, for two independent SO_3_(OH) tetra­hedra; the SO_4_ group in the thorium com­pound has a mean S—O bond length of 1.467 Å.

In the crystal structure of (NH_4_)Mg(HSO_4_)(SO_4_)(H_2_O)_2_, the short hydrogen bond between the S2O_3_(OH) and the S1O_4_ tetra­hedra [O8⋯O4^iii^ = 2.5048 (12) Å; Table 4 ▸] is linear [177 (3)°] and considered as strong (Jeffrey, 1997 ▸). In com­parison, the other types of O—H⋯O hydrogen-bonding inter­actions are much weaker and are connected with the two water mol­ecules. One of the water mol­ecules (O9) is involved in a slightly bent hydrogen bond of medium strength to atom O1^iv^ and in a weak trifurcated hydrogen bond to O2^i^, O5^v^ and O7^v^; numerical values of these inter­actions, as well as symmetry codes, are collated in Table 4 ▸. The other water mol­ecule (O10) is the donor of one medium–strong and slightly bent hydrogen bond to O5^vii^, and of a weak bifurcated hydrogen bond to O1^vi^ and O2^vi^. As expected, the ammonium cation is also engaged in hydrogen bonding. All of its H atoms are accepted in a more or less linear manner [N—H⋯O angles range from 168.2 (15) to 179.2 (18)°] by the O atoms of the sulfate group (O4^v^, O1^i^ and O3^iii^) and, inter­estingly, by the OH group of the hydrogen sulfate anion (O8^viii^). The latter hydrogen bond is much more bent [154.4 (16)°], most probably due to steric reasons to avoid a too close contact with the H atom of the hy­droxy group.

Bond-valence sums (BVSs; Brown, 2002 ▸), calculated with the parameters of Brese & O’Keeffe (1991 ▸), amount to 2.22 valence units (v.u.) for Mg, 5.98 v.u. for S1 and 5.96 v.u. for S2, in good agreement with the formal charges of +II and +VI, respectively.

#### Phase transition   

**Figure 4:**
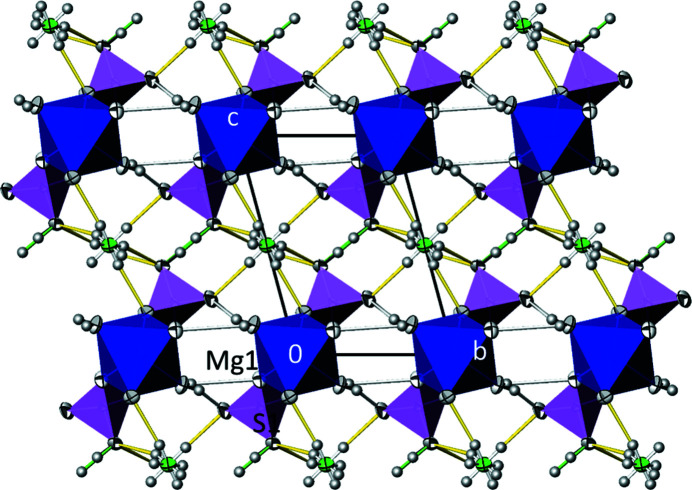
The crystal structure of (NH_4_)MgH(SO_4_)_2_(H_2_O)_2_ at 296 K in a projection along [100]. Displacement ellipsoids and colour codes are as in Fig. 2 ▸, except for the SO_4_ tetra­hedra which are lilac. The disordered ammonium group and the H1O atom disordered between two sulfate tetra­hedra are shown.

As mentioned above, at 100 K, (NH_4_)Mg(HSO_4_)(SO_4_)(H_2_O)_2_ crystallizes in an own structure type, denoted as **E1**. Between 100 K and room temperature, the crystal is transformed into a triclinic structure corresponding to type **E** (space group *P* 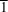, *Z* = 1) in the classification of com­pounds with kröhnkite-type chains (Table 1 ▸). Next to the six isotypic sulfates KFeH(SO_4_)_2_(H_2_O)_2_ (Fleck *et al.*, 2002*b* ▸), KMgH(SO_4_)_2_(H_2_O)_2_ (Macíček *et al.*, 1994 ▸), KZnH(SO_4_)_2_(H_2_O)_2_, KMnH(SO_4_)_2_(H_2_O)_2_, CsMnH(SO_4_)_2_(H_2_O)_2_ (Troy­anov *et al.*, 2002 ▸) and NH_4_FeH(SO_4_)_2_(H_2_O)_2_ (Heinicke *et al.*, 2004 ▸), and the selenate KMgH(SeO_4_)_2_(H_2_O)_2_ (Troyanov *et al.*, 2002 ▸), (NH_4_)Mg(HSO_4_)(SO_4_)(H_2_O)_2_, or more precisely (NH_4_)MgH(SO_4_)_2_(H_2_O)_2_ at this temperature, is the eighth member of this structure type. The [*M* ^II^O_4_(OH_2_)_2_] octa­hedron in these structures (Fig. 4 ▸ and Table 5 ▸) is located on an inversion centre, just like the *A* cation (for *A* = NH_4_; the H sites are disordered). A peculiarity of type **E** pertains to the dynamically disordered H atom between two symmetry-related sulfate groups, defining a short asymmetrical hydrogen bond with O⋯O contacts around 2.5 Å (Table 6 ▸). In com­parison, in the crystal structure of (NH_4_)Mg(HSO_4_)(SO_4_)(H_2_O)_2_ at 100 K, the H atom is ordered between two sulfate tetra­hedra, defining distinct SO_3_OH and SO_4_ groups. This ordering is accom­panied by a doubling of the unit-cell volume of the type **E1** relative. The bond lengths of the principal building units in the disordered room-temperature structure (Table 5 ▸; mean values for the Mg—O and S—O bond are 2.065 and 1.474 Å, respectively) are similar to those in the ordered low-temperature structure. Although the S—O(H) bond (O1) in the disordered structure is still the longest in the SO_4_ tetra­hedron, it is about 0.03 Å shorter than the S—OH bond (O8) in the ordered structure. On the other hand, the O1⋯O1^iv^ distance of the hydrogen bond with the disordered H1O atom [2.4790 (12) Å] is considerably shorter than the corresponding value in the ordered structure [2.5048 (12) Å], indicating a very strong hydrogen bond (Jeffrey, 1997 ▸) for (NH_4_)MgH(SO_4_)_2_(H_2_O)_2_.

The crystal structure of (NH_4_)Mg(HSO_4_)(SO_4_)(H_2_O)_2_ at 100 K represents a twofold superstructure with ordered H atoms for the ammonium and hydrogen sulfate groups relative to the crystal structure of (NH_4_)MgH(SO_4_)_2_(H_2_O)_2_ at 296 K with a halved unit-cell volume. The subcell of the latter is related to the doubled cell of the (NH_4_)Mg(HSO_4_)(SO_4_)(H_2_O)_2_ superstructure by application of the matrix (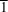 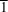 0, 1 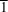 0, 0 0 1); the symmetry relationship between the substructure and the superstructure is of isomorphic type with index 2 (i2) (Müller, 2013 ▸). Fig. 1 ▸ shows the *hk*1 plane of reciprocal space of (NH_4_)Mg(HSO_4_)(SO_4_)(H_2_O)_2_ and the relation of the subcell (Fig. 1 ▸ *a*) and the cell of the actual superstructure (Fig. 1 ▸ *b*); the missing reflections for the subcell clearly indicate that the doubled cell is correct at this temperature. Fig. 1 ▸(*c*) shows the *hk*1 plane of reciprocal space of (NH_4_)MgH(SO_4_)_2_(H_2_O)_2_ without noticeable superstructure reflections for the room-temperature data set. Investigations of the exact ordering temperatures for this reversible phase transition upon cooling and heating, as well as a systematic study of other (NH_4_)*M* ^II^(HSO_4_)(SO_4_)(H_2_O)_2_ 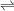 (NH_4_)*M* ^II^H(SO_4_)_2_(H_2_O)_2_ (*M* = first-row transition metals) phases, are underway.

#### IR spectroscopy   

**Figure 5:**
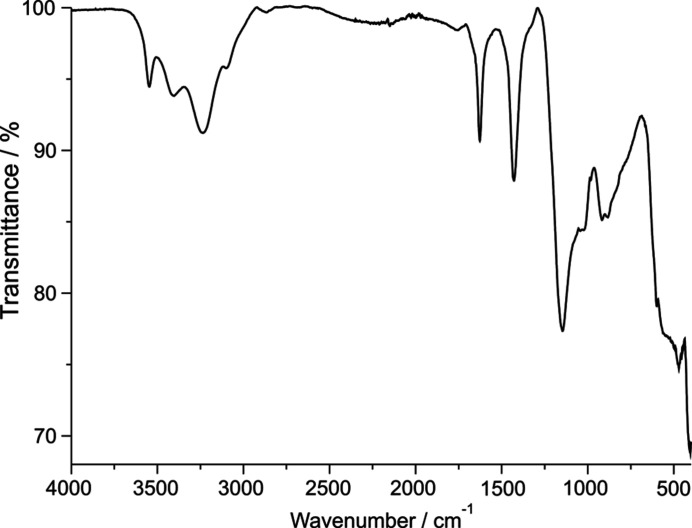
IR spectrum of (NH_4_)MgH(SO_4_)_2_(H_2_O)_2_ at room temperature.

The IR spectrum of (NH_4_)MgH(SO_4_)_2_(H_2_O)_2_ shows similarities to that of synthetic boussingaultite (Jayakumar *et al.*, 1988 ▸) and is displayed in Fig. 5 ▸. Wavenumbers/cm^−1^: 3547 (*w*), 3403 (*br*), 3235 (*br*), 3100 (*w*), 2866 (*vw*), 1753 (*vw*), 1627 (*m*), 1429 (*m*), 1146 (*s*), 1044 (*sh*), 918 (*m*), 884 (*m*), ≃600 (*sh*) (*br* = broad; *m* = medium; *s* = strong; *sh* = shoulder; *w* = weak; *vw* = very weak).

In the wavenumber range 3700–2500 cm^−1^, bands due to O—H stretching vibrations of the H_2_O groups overlap with various bands of the NH_4_ group. The bands at 3547 and 3403 cm^−1^ are assigned to the O—H stretching vibrations, while the band at 2335 cm^−1^ is tentatively assigned to the ν_3_(NH_4_) stretching vibration, and the shoulder at 3100 cm^−1^ to the ν_1_(NH_4_) stretch, the shoulder possibly also to an additional combination band ν_2_ + ν_4_(NH_4_). The very small band at 2866 cm^−1^ is probably caused by a combination band 2ν_4_(NH_4_). The very strong hydrogen bonding involving the protonated {H(SO_4_)_2_} group is reflected by an extremely broad band in the range between roughly 1200 and 1000 cm^−1^ (Beran & Libowitzky, 1999 ▸; Libowitzky, 1999 ▸), which appears ‘hidden’ in the background. The wavenumber range between 1800 and 1250 cm^−1^ contains bands due to the ν_2_(NH_4_) bending vibration (1627 cm^−1^, possibly also the very small band at 1753 cm^−1^) and the ν_4_(NH_4_) bending vibration (1429 cm^−1^). The range 1250–700 cm^−1^ shows bands due to vibrations of the SO_4_/HSO_4_ groups. The band at 1146 cm^−1^ is due to the ν_3_(SO_4_) stretching vibration, while the bands at 1044, 918 and 884 cm^−1^ are assigned to the ν_1_(SO_4_) stretching vibration. The shoulder at ∼600 cm^−1^ is problably due to the ν_4_(SO_4_) vibration. The ν_2_(SO_4_) bending vibration will cause bands <500 cm^−1^, where the spectrum is cut off and where bands due to vibrations of the MgO_6_ octa­hedron, the librational modes of the NH_4_ group and lattice modes are expected. Note that the presence of ‘forbidden’ SO_4_ and NH_4_ vibrations in the IR spectrum is in agreement with the presence of distorted shapes for these two building units.

### NaSc(CrO_4_)_2_(H_2_O)_2_   

#### Structure analysis   

**Figure 6:**
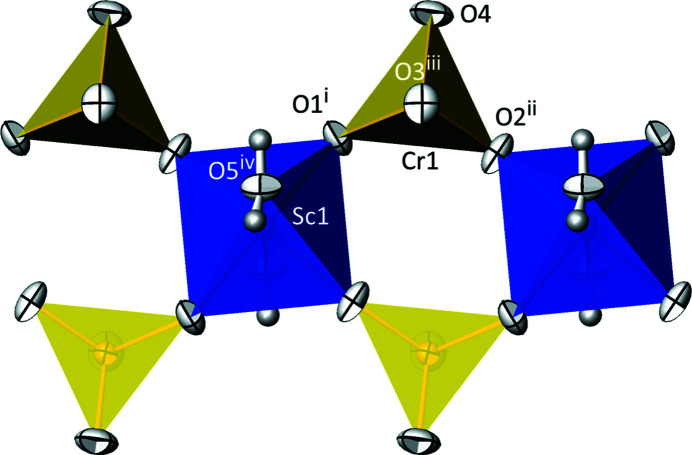
The kröhnkite-type chain in the crystal structure of NaSc(CrO_4_)_2_(H_2_O)_2_. [ScO_4_(OH_2_)_2_] octa­hedra are blue and CrO_4_ tetra­hedra are yellow. Displacement ellipsoids are drawn at the 74% probability level and H atoms are given as grey spheres of arbitrary radius. [Symmetry codes: (i) *x*, −*y* + 1, *z* + 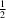; (ii) *x*, *y* + 1, *z*; (iii) *x*, −*y* + 1, *z* − 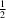; (iv) −*x* + 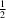, −*y* + 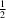, −*z*.]

**Figure 7:**
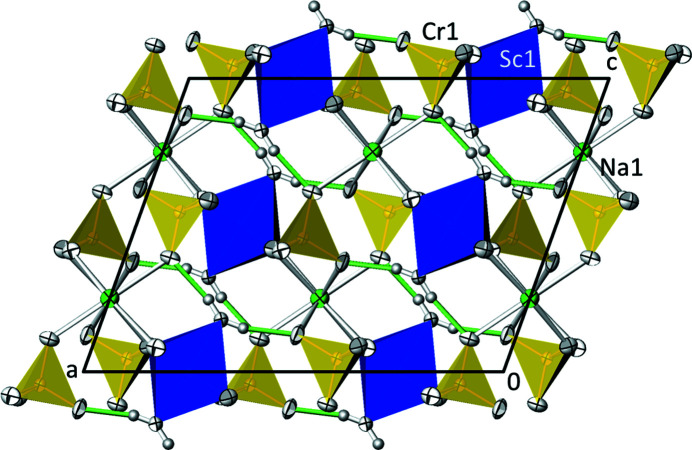
The crystal structure of NaSc(CrO_4_)_2_(H_2_O)_2_ in a projection along [010]. Colour codes and the probability level of displacement ellipsoids are as in Fig. 3 ▸. Hydrogen bonds are indicated as green lines.

NaSc(CrO_4_)_2_(H_2_O)_2_ adopts type **F** (subtype **F1**) of the classification scheme for structures with kröhnkite-type chains (Table 1 ▸). Subtype **F1** (space group *C*2/*c*, *Z* = 2) can be considered as a superstructure of subtype **F2** (space group *C*2/*m*, *Z* = 1) that has a halved unit-cell volume relative to **F1** [transformation matrix F1→F2 is (0 0 1, 0 1 0, 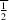 0 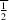)]. The group–subgroup relationship between subtypes **F2** and **F1** is klassengleich with index 2 (k2) (Müller, 2013 ▸). In the crystal structure of NaSc(CrO_4_)_2_(H_2_O)_2_, [ScO_4_(OH_2_)_2_] octa­hedra (point-group symmetry 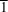) are linked by CrO_4_ tetra­hedra into chains running parallel to [010] (Fig. 6 ▸). The Na^I^ cations (site symmetry 2) connect adjacent chains into a three-dimensional framework that is stabilized by hydrogen bonds between water mol­ecules and sulfate O atoms (Fig. 7 ▸).

In the [ScO_4_(OH_2_)_2_] octa­hedron, the longest bond [2.1222 (14) Å] is that to the axially bound O5 atom of the water mol­ecule, whereas the equatorial O atoms (O3 and O4), which are also part of a CrO_4_ tetra­hedron, have shorter Sc—O bonds, with a mean of 2.076 Å (Table 7 ▸). The overall mean value for the Sc—O bond lengths is 2.091 Å, which matches very well the literature values of 2.10 (7) and 2.098 (41) Å given by Serezhkin *et al.* (2003 ▸) and Gagné & Hawthorne (2020 ▸), respectively. In the CrO_4_ tetra­hedron, the longest Cr—O bonds (≃1.69 Å) are realized for O1 and O2, which are part of the kröhnkite chains. The other two O atoms (O3 and O4) have considerably shorter Cr—O bonds (≃1.62 Å) and are the acceptor atoms for two nearly linear hydrogen bonds of medium–strong nature involving both water H atoms (Table 8 ▸). Again, the mean Cr—O bond length of 1.651 Å is in very good agreement with the literature value of 1.65 (6) Å (Gagné & Hawthorne, 2020 ▸). The Na^I^ cation shows a [6 + 2] coordination with the six closer O atoms defining a distorted octa­hedron (O1, O2 and their symmetry-related counterparts in equatorial sites, and O3 and its symmetry-related counterpart in axial sites), with the two remote O4 atoms capping two faces of the octa­hedron (Table 7 ▸). Notably, the water mol­ecule is not part of the coordination sphere of Na. The mean Na—O bond length is 2.678 Å, somewhat longer than that of the literature value of 2.60 (19) Å for eightfold-coordinated Na^I^ (Gagné & Hawthorne, 2016 ▸). This elongation is also reflected in the slight underbonding of Na1 in the structure (BVS = 0.83 v.u.), with a deviation of 17% from the expected value of +I. Sc^III^ and Cr^VI^, on the other hand, have BVS values of 3.12 and 5.92 v.u., respectively, and deviate much less (by about 4 and 2%) from the expected values.

NaSc(CrO_4_)_2_(H_2_O)_2_ is isotypic with NaAl(CrO_4_)_2_(H_2_O)_2_ (Cudennec & Riou, 1977 ▸) and NaFe(CrO_4_)_2_(H_2_O)_2_ (Hardy & Gravereau, 1970 ▸), the only other members of structure type **F1** in the classification of structures with kröhnkite-type chains. The title scandium com­pound is the first of this series for which the H atoms have been localized, thus making an unambiguous assignment of the hydrogen-bonding scheme possible (see above). For a qu­anti­tative structural com­parison of the three isotypic Na*M*(CrO_4_)_2_(H_2_O)_2_ (*M* = Sc, Al or Fe) structures, the program *com­pstru* (de la Flor *et al.*, 2016 ▸), available at the Bilbao Crystallographic Server (Aroyo *et al.*, 2006 ▸), was used. With NaSc(CrO_4_)_2_(H_2_O)_2_ as the reference structure, Table 9 ▸ com­piles the absolute distances between paired atoms and numerical values regarding the arithmetic mean of the distance between paired atoms, the degree of lattice distortion (Δ) and the measure of similarity (S). There is no clear trend as to the largest displacement of an atom pair in the three crystal structures. Whereas the water O atom (O5) in the *M* = Al structure shows the largest displacement, it is O1 in the *M* = Fe structure. In general, the rather low values of S indicate high similarities between NaSc(CrO_4_)_2_(H_2_O)_2_ and the two Na*M*(CrO_4_)_2_(H_2_O)_2_ (*M* = Al and Fe) structures, whereby the *M* = Fe structure has a higher absolute similarity to NaSc(CrO_4_)_2_(H_2_O)_2_. Most likely, this behaviour is related to the ionic radii (Shannon, 1976 ▸) of the three *M* ^III^ cations. For coordination number 6, the ionic radius of Sc^III^ (0.745 Å) is closer to that of Fe^III^ (0.645 Å, assuming a high-spin state) than to that of Al^III^ (0.535 Å).

## Supplementary Material

Crystal structure: contains datablock(s) NH4MgHSO4SO4H2O2_100K, NH4MgHSO42H2O_296K, NaScCrO42H2O2, global. DOI: 10.1107/S2053229621001650/ef3013sup1.cif

Structure factors: contains datablock(s) NH4MgHSO4SO4H2O2_100K. DOI: 10.1107/S2053229621001650/ef3013NH4MgHSO4SO4H2O2_100Ksup2.hkl

Structure factors: contains datablock(s) NH4MgHSO42H2O_296K. DOI: 10.1107/S2053229621001650/ef3013NH4MgHSO42H2O_296Ksup3.hkl

Structure factors: contains datablock(s) NaScCrO42H2O2. DOI: 10.1107/S2053229621001650/ef3013NaScCrO42H2O2sup4.hkl

CCDC references: 2062574, 2062573, 2062572

## Figures and Tables

**Table 1:** Comparative com­pilation of structure types with kröhnkite-type chains

Type	Generalized formula(e)	Space group	*Z*	Generalized unit-cell parameters (Å, °)	No. of representatives
**A**	*M*1^II^_2_ *M*2^II^(*T*O_4_)_2_(H_2_O)_2_: *M*1 = Ca, Sr; *M* ^II^ = Mg, Mn, Fe, Co, Ni, Zn; *T* = P, As. *M*1^I^_2_ *M*2^II^(*T*O_4_)_2_(H_2_O)_2_: *M*1 = Na, K, NH_4_; *M* ^II^ = Mg, Fe, Co, Ni, Cu, Zn; *T* = S, Se, Cr, Mo, W	*P*\overline{1}	1	*a* ≃ 5.7–7.1, *b* ≃ 6.7–7.9, *c* ≃ 5.3–6.0, α ≃ 93–105, β ≃ 105–112, γ ≃ 103–111	32
**B**	*M*1^II^_2_ *M*2^II^(*T*O_4_)_2_(H_2_O)_2_: *M*1 = Ca; *M*2 = Mn, Fe; *T* = P	*P*\overline{1}	1	*a* ≃ 5.8–6.0, *b* ≃ 6.5–6.6, *c* ≃ 5.5, α ≃ 102–103, β ≃ 108–109, γ ≃ 90–91	2
**C**	*M*1^I^_2_ *M*2^II^(*T*O_4_)_2_(H_2_O)_2_: *M*1 = K, Rb; *M*2 = Mn, Cd; *T* = S, Se, Cr	*P*\overline{1}	2	*a* ≃ 6.6–6.9, *b* ≃ 7.3–7.7, *c* ≃ 10.7–11.4, α ≃ 72–73, β ≃ 74–75, γ ≃ 70	4
**C1**	K_2_Fe(SO_4_)_2_(H_2_O)_2_	*P*1^*a*^	2	*a* ≃ 6.6, *b* ≃ 7.3, *c* ≃ 10.7, α ≃ 73, β ≃ 74, γ ≃ 70	1
**D**	*M*1^II^_2_ *M*2^II^(*T*O_4_)_2_(H_2_O)_2_: *M*1 = Ca; *M*2 = Mg, Mn, Co, Cu, Zn; *T* = As. *M*1^I^_2_ *M*2^II^(*T*O_4_)_2_(H_2_O)_2_: *M*1 = Na, NH_4_, Rb; *M*2 = Cr, Mn, Fe, Cu, Cd; *T* = S, Se, Cr, Mo	*P*2_1_/*c*	2	*a* ≃ 5.8–6.8, *b* ≃ 12.8–14.3, *c* ≃ 5.4–5.9, β ≃ 106–111	14
**E**	*M*1^I^ *M*2^II^H(*T*O_4_)_2_(H_2_O)_2_: *M*1 = K, NH_4_, Cs; *M*2 = Mg, Mn, Fe, Co, Zn; *T* = S, Se	*P*\overline{1}	1	*a* ≃ 4.6–4.8, *b* ≃ 5.7–5.9, *c* ≃ 8.1–8.6, α ≃ 103–104, β ≃ 96–100, γ ≃ 94–97	8
**E1** ^*b*^	(NH_4_)Mg(HSO_4_)(SO_4_)(H_2_O)_2_ (at 100 K)	*P*\overline{1}	2	*a* ≃ 7.1, *b* ≃ 7.7, *c* ≃ 8.3, α ≃ 84.6, β ≃ 73.3, γ ≃ 77.4	1
**F1** ^*c*^	*M*1^I^ *M*2^III^(*T*O_4_)_2_(H_2_O)_2_: *M*1 = Na; *M*2 = Al, Sc, Fe; *T* = Cr	*C*2/*c*	4	*a* ≃ 14.1–14.5, *b* ≃ 5.3–5.6, *c* ≃ 10.7–10.8, β ≃ 109–110	3
**F2** ^*c*^	*M*1^I^ *M*2^III^(*T*O_4_)_2_(H_2_O)_2_: *M*1 = Na, K, NH_4_, Tl; *M*2 = Al, Fe, In; *T* = Cr	*C*2/*m*	2	*a* ≃ 10.7–11.0, *b* ≃ 5.4–5.6, *c* ≃ 7.5–7.6, β ≃ 114	5
**G**	AgSc(CrO_4_)_2_(H_2_O)_2_	*P*\overline{1}	1	*a* ≃ 5.6, *b* ≃ 6.1, *c* ≃ 7.4, α ≃ 111, β ≃ 90, γ ≃ 117	1
**H** ^*d*^	K_2_Zn(CrO_4_)_2_(H_2_O)_2_	*C*2/*c*	4	*a* ≃ 15.0, *b* ≃ 5.7, *c* ≃ 12.3, β ≃ 117	1

Notes: (*a*) Type C1 is represented only by the low-temperature modification of K_2_Fe(SO_4_)_2_(H_2_O)_2_ and has an uncertain space group. (*b*) Type **E1** is described for the first time in the present work; it is an ordered variant of type **E** (see text). (*c*) Types **F1** and **F2** are closely related (see text). (*d*) Type **H** (Stoilova *et al.*, 2008 ▸) is closely related to type **A**.

**Table 2:** Experimental details Experiments were carried out with Mo *K*α radiation. All H-atom parameters were refined.

	**(NH_4_)Mg(HSO_4_)(SO_4_)(H_2_O)_2_ at 100 K**	**(NH_4_)MgH(SO_4_)_2_(H_2_O)_2_ at 296 K**	**NaSc(CrO_4_)_2_(H_2_O)_2_**
Crystal data			
Chemical formula	(NH_4_)Mg(HSO_4_)(SO_4_)(H_2_O)_2_	(NH_4_)Mg(HSO_4_)(SO_4_)(H_2_O)_2_	NaSc(CrO_4_)_2_(H_2_O)_2_
*M*_r_	271.51	271.51	335.98
Crystal system, space group	Triclinic, *P*\overline{1}	Triclinic, *P*\overline{1}	Monoclinic, *C*2/*c*
Temperature (K)	100	296	293
*a*, *b*, *c* (Å)	7.0631 (7), 7.7065 (7), 8.3372 (8)	4.6771 (1), 5.7697 (1), 8.3697 (2)	14.505 (3), 5.563 (1), 10.763 (2)
α, β, γ (°)	84.603 (3), 73.339 (3), 77.387 (3)	104.208 (1), 98.189 (1), 94.508 (1)	90, 109.82 (3), 90
*V* (Å^3^)	424.03 (7)	215.20 (1)	817.0 (3)
*Z*	2	1	4
μ (mm^−1^)	0.75	0.73	3.51
Crystal size (mm)	0.12 × 0.09 × 0.02	0.12 × 0.09 × 0.01	0.17 × 0.10 × 0.03

Data collection			
Diffractometer	Bruker APEXII CCD	Bruker APEXII CCD	Nonius KappaCCD
Absorption correction	Multi-scan (*SADABS*; Krause *et al.*, 2015 ▸)	Multi-scan (*SADABS*; Krause *et al.*, 2015 ▸)	Multi-scan (*SCALEPACK*; Otwinowski *et al.*, 2003 ▸)
*T*_min_, *T*_max_	0.708, 0.747	0.699, 0.747	0.755, 0.949
No. of measured, independent and observed [*I* > 2σ(*I*)] reflections	30998, 4431, 3120	11147, 1940, 1843	3418, 1783, 1372
*R*_int_	0.051	0.024	0.017
(sin θ/λ)_max_ (Å^−1^)	0.864	0.812	0.805

Refinement			
*R*[*F* ^2^ > 2σ(*F* ^2^)], *wR*(*F* ^2^), *S*	0.033, 0.081, 1.02	0.019, 0.054, 1.18	0.027, 0.081, 1.08
No. of reflections	4431	1940	1783
No. of parameters	163	95	75
No. of restraints	0	0	2
Δρ_max_, Δρ_min_ (e Å^−3^)	0.52, −0.54	0.30, −0.47	1.06, −0.69

Computer programs: *APEX2* (Bruker, 2016 ▸), *COLLECT* (Nonius, 2003 ▸), *DENZO* and *SCALEPACK* (Otwinowski *et al.*, 2003 ▸), *SHELXT* (Sheldrick, 2015*a* ▸), *SHELXS97* (Sheldrick, 2008 ▸), *SHELXL2018* (Sheldrick, 2015*b* ▸), *ATOMS* (Dowty, 2006 ▸) and *publCIF* (Westrip, 2010 ▸).

**Table 3:** Selected bond lengths (Å) for (NH_4_)Mg(HSO_4_)(SO_4_)(H_2_O)_2_ at 100 K

Mg1—O2	2.0382 (9)	S1—O2	1.4685 (8)
Mg1—O7	2.0601 (9)	S1—O3	1.4775 (8)
Mg1—O3^i^	2.0630 (9)	S1—O4	1.4901 (9)
Mg1—O10	2.0645 (9)	S2—O5	1.4480 (9)
Mg1—O9	2.0660 (10)	S2—O6	1.4583 (8)
Mg1—O6^ii^	2.0715 (9)	S2—O7	1.4611 (8)
S1—O1	1.4659 (9)	S2—O8	1.5474 (9)

Symmetry codes: (i) -x, -y+1, -z+1; (ii) -x+1, -y, -z+1.

**Table 4:** Hydrogen-bond geometry (Å, °) for (NH_4_)Mg(HSO_4_)(SO_4_)(H_2_O)_2_ at 100 K

*D*—H⋯*A*	*D*—H	H⋯*A*	*D*⋯*A*	*D*—H⋯*A*
O8—H1O⋯O4^iii^	0.91 (3)	1.59 (3)	2.5048 (12)	177 (3)
O9—H1*W*⋯O1^iv^	0.81 (2)	2.02 (2)	2.7623 (13)	154 (2)
O9—H2*W*⋯O2^i^	0.78 (3)	2.56 (3)	3.1278 (12)	132 (2)
O9—H2*W*⋯O5^v^	0.78 (3)	2.62 (3)	3.2887 (12)	146 (2)
O9—H2*W*⋯O7^v^	0.78 (3)	2.55 (3)	2.9578 (12)	115 (2)
O10—H3*W*⋯O1^vi^	0.77 (2)	2.38 (2)	3.0970 (12)	156 (2)
O10—H3*W*⋯O2^vi^	0.77 (2)	2.64 (2)	3.0615 (12)	117 (2)
O10—H4*W*⋯O5^vii^	0.80 (2)	1.95 (2)	2.7125 (13)	160.5 (19)
N1—H1N⋯O4^v^	0.896 (19)	2.108 (19)	2.9913 (14)	168.2 (15)
N1—H2N⋯O1^i^	0.85 (2)	2.04 (2)	2.8920 (14)	179.2 (18)
N1—H3N⋯O3^iii^	0.868 (19)	1.98 (2)	2.8451 (14)	170.5 (18)
N1—H4N⋯O8^viii^	0.870 (19)	2.229 (19)	3.0368 (14)	154.4 (16)

Symmetry codes: (i) -x, -y+1, -z+1; (iii) x+1, y, z-1; (iv) x+1, y, z; (v) -x+1, -y+1, -z+1; (vi) -x, -y, -z+1; (vii) x-1, y, z; (viii) x, y+1, z.

**Table 5:** Selected bond lengths (Å) for (NH_4_)MgH(SO_4_)_2_(H_2_O)_2_ at 296 K

Mg1—O2	2.0509 (6)	S1—O2	1.4604 (6)
Mg1—O4^i^	2.0720 (6)	S1—O4	1.4651 (6)
Mg1—O5	2.0731 (6)	S1—O1	1.5164 (6)
S1—O3	1.4525 (6)		

Symmetry code: (i) x+1, y, z.

**Table 6:** Hydrogen-bond geometry (Å, °) for (NH_4_)MgH(SO_4_)_2_(H_2_O)_2_ at 296 K

*D*—H⋯*A*	*D*—H	H⋯*A*	*D*⋯*A*	*D*—H⋯*A*
O5—H5*B*⋯O2^ii^	0.81 (2)	2.52 (2)	3.0010 (9)	118.9 (19)
O5—H5*A*⋯O3^iii^	0.78 (2)	2.05 (2)	2.7684 (9)	154.0 (19)
O5—H5*B*⋯O3^ii^	0.81 (2)	2.42 (2)	3.1678 (10)	152 (2)
N1—H1*A*⋯O3^iv^	0.90 (5)	2.03 (5)	2.9260 (6)	175 (5)
N1—H1*C*⋯O1	0.89 (6)	2.28 (6)	3.1536 (7)	164 (4)
N1—H1*B*⋯O1^v^	0.83 (5)	2.30 (5)	3.1122 (8)	164 (5)
N1—H1*D*⋯O4^v^	0.82 (5)	2.08 (5)	2.9042 (6)	173 (5)
O1—H1O⋯O1^iv^	0.84 (3)	1.64 (3)	2.4790 (12)	176 (4)

Symmetry codes: (ii) -x, -y-1, -z; (iii) -x-1, -y-1, -z; (iv) -x-1, -y-1, -z-1; (v) -x-1, -y, -z-1.

**Table 7:** Selected bond lengths (Å) for NaSc(CrO_4_)_2_(H_2_O)_2_

Na—O3^i^	2.5201 (15)	Sc—O5	2.1222 (14)
Na—O2^i^	2.5358 (15)	Cr—O4	1.6045 (14)
Na—O1^ii^	2.7207 (17)	Cr—O3^iv^	1.6204 (14)
Na—O4^i^	2.9360 (18)	Cr—O1^iv^	1.6829 (12)
Sc—O1^iii^	2.0747 (12)	Cr—O2^v^	1.6960 (11)
Sc—O2	2.0772 (12)		

Symmetry codes: (i) -x+{\script{1\over 2}}, y+{\script{1\over 2}}, -z+{\script{1\over 2}}; (ii) x-{\script{1\over 2}}, -y+{\script{3\over 2}}, z-{\script{1\over 2}}; (iii) -x+{\script{1\over 2}}, y-{\script{1\over 2}}, -z+{\script{1\over 2}}; (iv) x, -y+1, z-{\script{1\over 2}}; (v) x, y+1, z.

**Table 8:** Hydrogen-bond geometry (Å, °) for NaSc(CrO_4_)_2_(H_2_O)_2_

*D*—H⋯*A*	*D*—H	H⋯*A*	*D*⋯*A*	*D*—H⋯*A*
O5—H1⋯O4^vi^	0.82 (3)	1.86 (3)	2.682 (2)	179 (3)
O5—H2⋯O3	0.82 (2)	1.97 (3)	2.765 (2)	164 (3)

Symmetry code: (vi) x-{\script{1\over 2}}, y-{\script{1\over 2}}, z.

**Table 9:** Absolute atomic displacements (Å), arithmetic mean (*d*_av_, Å), degree of lattice distortion (S) and measure of similarity (Δ) in the isotypic Na*M*(CrO_4_)(H_2_O)_2_ (*M* = Al and Fe) structures relative to NaSc(CrO_4_)_2_(H_2_O)_2_^*a*^

	*M* = Al^*b*^	*M* = Fe^*c*^
Na1	0.0595	0.0773
*M*1	0	0
Cr1	0.0839	0.0536
O1	0.1477	0.1608
O2	0.1227	0.1436
O3	0.0595	0.0395
O4	0.0395	0.0520
O5	0.1916	0.0751
		
*d*_av_	0.0964	0.0805
Δ	0.023	0.019
S	0.0186	0.0107

Notes: (*a*) H atoms were omitted from com­parison because in the *M* = Al and Fe structures, H atoms were not localized. (*b*) Unit-cell parameters *a* = 14.080 (10), *b* = 5.338 (3), *c* = 10.655 (6) Å and β = 110.33 (5)° (Cudennec & Riou, 1977 ▸). (*c*) Unit-cell parameters *a* = 14.247 (2), *b* = 5.425 (5), *c* = 10.689 (2) Å and β = 109.30 (1)° (Hardy & Gravereau, 1970 ▸).
